# Framework for In-Field Analyses of Performance and Sub-Technique Selection in Standing Para Cross-Country Skiers

**DOI:** 10.3390/s21144876

**Published:** 2021-07-17

**Authors:** Camilla H. Carlsen, Julia Kathrin Baumgart, Jan Kocbach, Pål Haugnes, Evy M. B. Paulussen, Øyvind Sandbakk

**Affiliations:** 1 Centre for Elite Sports Research, Department of Neuromedicine and Movement Science, Faculty of Medicine and Health Sciences, Norwegian University of Science and Technology, 7491 Trondheim, Norway; julia.k.baumgart@ntnu.no (J.K.B.); jan.kocbach@ntnu.no (J.K.); pal.haugnes@ntnu.no (P.H.); Evy.paulussen@mumc.nl (E.M.B.P.); oyvind.sandbakk@ntnu.no (Ø.S.); 2 NORCE Norwegian Research Centre AS, 5008 Bergen, Norway; 3 Faculty of Health, Medicine & Life Sciences, Maastricht University, 6200 MD Maastricht, The Netherlands

**Keywords:** micro-sensor technology, GNSS, IMU, disability, heterogenous group, cross-country skiing race, performance analysis, sub-technique classification, time factor

## Abstract

Our aims were to evaluate the feasibility of a framework based on micro-sensor technology for in-field analyses of performance and sub-technique selection in Para cross-country (XC) skiing by using it to compare these parameters between elite standing Para (two men; one woman) and able-bodied (AB) (three men; four women) XC skiers during a classical skiing race. The data from a global navigation satellite system and inertial measurement unit were integrated to compare time loss and selected sub-techniques as a function of speed. Compared to male/female AB skiers, male/female Para skiers displayed 19/14% slower average speed with the largest time loss (65 ± 36/35 ± 6 s/lap) found in uphill terrain. Female Para/AB skiers utilized DP, DK, and DIA, 61/43%, 15/10%, and 25/47% of the distance at low speeds, respectively, while the corresponding numbers for male Para/AB skiers were 58/18%, 1/13%, and 40/69%. At higher speeds, female Para/AB skiers utilized DP and OTHER, 26/52% and 74/48% of the distance, respectively, while corresponding numbers for male Para/AB skiers were 29/66% and 71/34%. This indicates different speed thresholds of the classical sub-techniques for Para than AB skiers. The framework provides a point of departure for large-scale international investigations of performance and related factors in Para XC skiing.

## 1. Introduction

Para cross-country (XC) skiing is a winter sport performed by skiers with different disabilities. Depending on their disability, Para XC skiers compete in three categories, which are further divided into classes, based on the functional impact of the disability on XC skiing performance: (1) physically impaired sitting skiers (classes: LW10–12), (2) physically impaired standing skiers (classes: LW2–9), and (3) visually impaired standing skiers (classes: B1–3) [1,2]. Additionally, within each category, a class-specific time factor is used to calculate the final race time [1,2].

Physically impaired standing XC skiers constitute a heterogenous group of skiers with different disabilities, which range from having an amputation to muscle weakness or loss of muscle control [1,2]. Similar to able-bodied (AB) XC skiers, standing Para XC skiers compete within the classical and skating styles in race courses consisting of undulating terrain with uphill, flat, and downhill segments [3]. The varying terrain during XC skiing races leads to substantial variation in speed, which is regulated by selection of pacing strategies, sub-techniques, and related kinematic patterns [4,5,6,7]. In the classical style, XC skiers alternate between double poling (DP), which is used at higher speeds on a wide range of inclines [8,9], kick double poling (DK), which is used at moderate speeds in the transition between different terrains [10], diagonal stride (DIA), which is primarily used at low speeds in moderate to steep uphill terrain [11,12], and the herringbone technique (HRB), which is used at low speeds in very steep uphill terrain [13]. During downhill sections, the skiers employ the tuck position without pole and leg actions, and various turn techniques are adapted to manage turning [4,14]. The choice of sub-technique and regulation of kinematic patterns is complex and influenced by individual preferences, internal, and external factors [6,11,12,15,16,17,18]. In classical AB XC skiing, it has been suggested that there are speed [6,17,18] and incline [11,12,16] thresholds for the use of the sub-techniques. Additionally, the skiers’ physical capacity will influence the speed and choice of sub-technique [19,20]. In this context, the ability to use the different sub-techniques may additionally be dependent on functional limitations related to the individual disability among standing Para XC skiers. Accordingly, the sub-technique selected at different speeds may differ between standing Para and AB XC skiers.

Related to determination of the above parameters, micro-sensor technology has allowed detailed in-field performance analyses with continuous speed and time tracking, as well as automatic sub-technique classification, and is widely used among AB XC skiers [6,15,21]. However, in standing Para XC skiing, analyses of in-field performance or sub-technique distribution have not yet been done. Accordingly, a framework for such analyses would be beneficial for providing new insights into the technical and tactical aspects, as well as the effect of terrain and external conditions on the time factor, related to standing Para XC skiing performance.

Therefore, the aim of this study was to evaluate the feasibility of a framework based on micro-sensor technology for detailed analyses of in-field performance and sub-technique selection in Para XC skiing by using this framework in case-series to descriptively compare performance-related parameters between elite standing Para and AB XC skiers during a classical skiing race.

## 2. Materials and Methods

### 2.1. Participants

Three elite standing Para XC skiers (two male B3 skiers, one female LW4 skier) of the Norwegian national team, and nine elite AB XC skiers (five men, four women) of the Norwegian B national team participated in the study (Table 1). The male B3 skiers had 10% vision and were accompanied by a personal guide during the race. The female LW4 skier had linear scleroderma with reduced leg length, joint mobility, muscle mass, and strength in the one leg. Due to a limited number of elite standing Para XC skiers within the same category, AB XC skiers were used as reference to evaluate the feasibility of the framework. Among the male AB XC skiers, there was one participant with missing data due to complications with tracking during the race and one with an unfinished race. Their data were omitted and data of three male and four female AB XC skiers were included in the analyses. All participants signed an informed consent form and were made aware that they could withdraw from the study at any point without providing an explanation. The study was approved by the Norwegian Centre for Research Data (ID 49865/3/IJJ) and conducted in line with the Declaration of Helsinki.

### 2.2. Design

**Figure 1:**
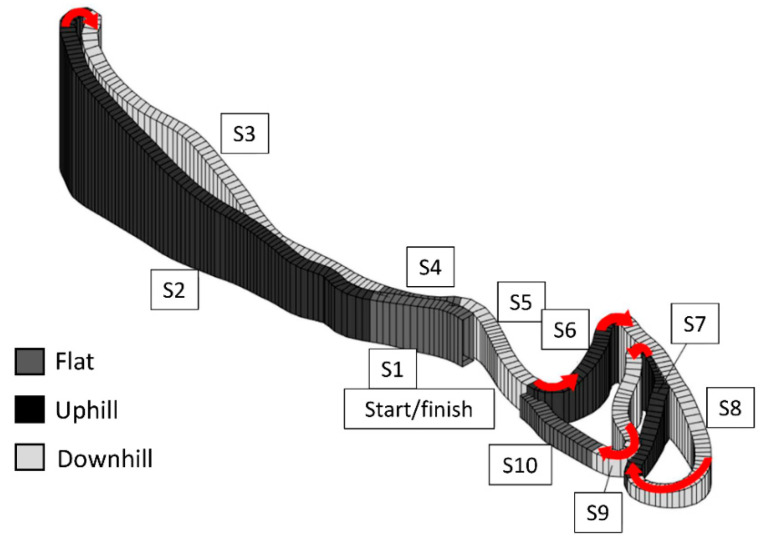
The 2.5 km XC skiing race course divided into the 10 segments according to the elevation difference, including three uphill, three flat, and four downhill segments. Six turns were distributed over the 2.5 km lap (red arrow). With different placement of the start and finish, there is a gap in the 2.5 km course, which was removed from the analyses. (S) Segment (length (meter), incline range (%)); S1: 131 m, −2.6–1.6%; S2: 543 m, 1.7–12.4%; S3: 509 m, −11.7–0.2%; S4 100 m, 0.7–2.8%; S5: 156 m, −6.0–0.0%; S6: 166 m, 1.3–12.7%; S7: 339 m, −8.5–−0.4%; S8: 200 m, 1.2–16%; S9: 183 m, −10.1–−1.0%; S10: 138 m, 0.0–1.6%.

During a national competition, participants performed a time-trial XC skiing race on snow using the classical style. The time-trial was performed on a 2.5 km race course, where female Para and AB XC skiers raced 10 km (4 × 2.5 km) and male Para and AB XC skiers raced 15 km (6 × 2.5 km), in accordance with the International Ski Federation regulations (Figure 1). During the race, each Para and AB XC skier was continuously tracked with a Catapult device (OptimEye S5, Catapult Innovations, Melbourne, Australia) with integrated 10 Hz global navigation satellite system (GNSS) receiver and an inertial measurement unit (IMU) providing 100 Hz triaxial accelerometer and gyroscope data, positioned in a tight fitting-vest on the skier’s upper back under the race bib. All XC skiers raced on the same day. The time-trials started in the morning, with the female AB XC skiers racing first followed by the male AB XC skiers. Thereafter, the female and male Para XC skiers completed their race within the same time range. The start interval between each Para and AB XC skier was 30–60 s. Every athlete used their own ski equipment, including skis, poles, boots, and ski base material (including grinds, structure, and waxing), with adjustments being made by each team’s waxing crew according to individual preferences and daily conditions. The weather conditions were stable throughout the day, with a snow temperature of −12 °C and an air temperature between −4 to −7 °C during all the races. The snow friction was measured as 0.023 in the middle of the day. The course was covered with hard-packed snow and machine-prepared directly before the races of the AB and Para XC skiers.

### 2.3. Measurements

Time, positioning, altitude, and movement data for all Para and AB XC skiers were measured continuously during the race with the Catapult devices. The speed data were derived from time differentiation of the position data. Prior to the data collection, the Catapult devices were placed outside in an open space for a minimum of 10 min to ensure GNSS lock and allow acquisition of satellite signals. Recently, Gløersen et al. [22] have validated the Catapult devices for position, speed, and time analyses in AB XC skiing against a geodetic, multi-frequency receiver, with a horizontal plane position error of 1.04 m (third quartile, Q3), horizontal plane speed of 0.072 m·s^−1^ (IQR), and time precision between 0.13–0.36 s. Similar accuracy is expected when using the Catapult devices for analysis of performance as done in the current study.

### 2.4. Data Analysis

**Figure 2:**
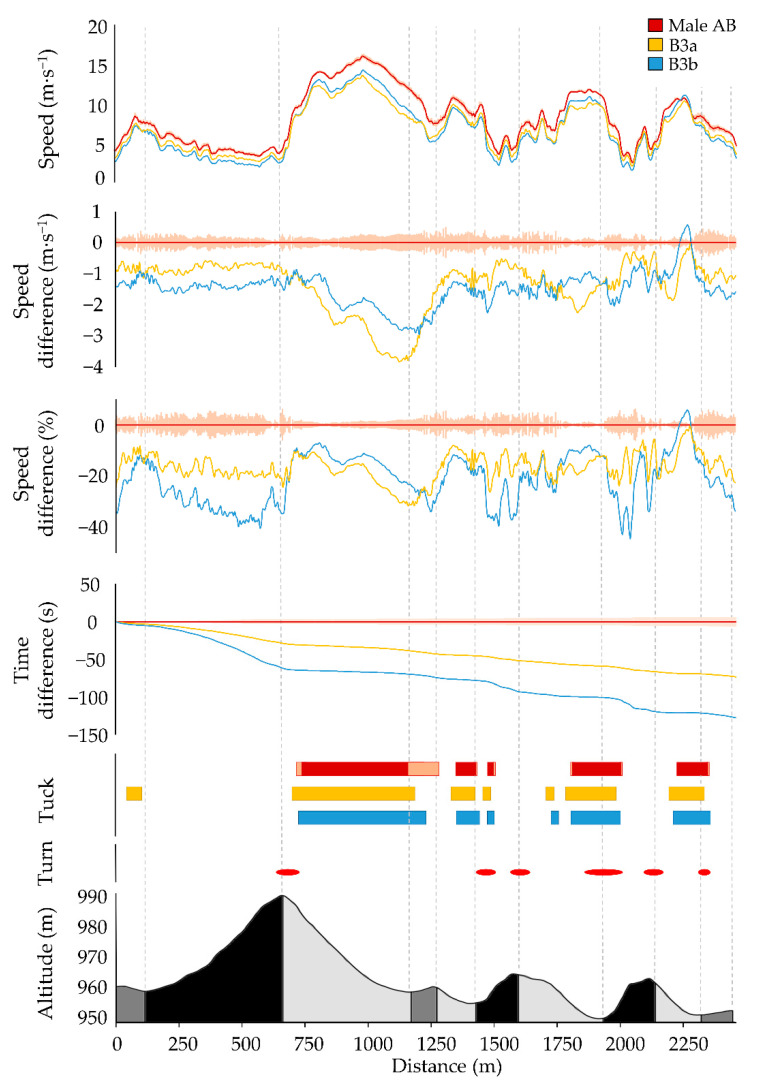
Comparison of average male AB XC skiers (dark red; standard deviation light pink), male B3a XC skier (yellow), and male B3b XC skier (blue) for the six laps during the race with respect to average speed, absolute speed difference, relative speed difference, accumulated time difference, and tuck. Course details are visualized in the lower part of the figure; turns (red dashes) and altitude profile of the 2.5 km race course, with uphill (black), flat (dark gray), and downhill (light gray) segments.

The length and elevation profile of the race course were obtained from the GNSS data measured with the Catapult devices that were used to track the Para and AB XC skiers. Based on the positioning and altitude data, the course was divided into segments consisting of either uphill, flat, and downhill terrain. Each segment began and ended with an evident change in the gradient of the course. The uphill and downhill segments were characterized by a minimum elevation difference of 4 m from the beginning to the end of the segment. Undulating terrain with a smaller elevation difference between adjacent uphill, flat, and downhill segments were merged into one single flat segment. Overall, the 2.5 km course was divided into 10 segments, with three uphill, three flat, and four downhill segments that made up 37%, 15%, and 48% of the course, respectively. Additionally, the 2.5 km course included six turns (Figure 2). Different placements of the start and finish of the race resulted in a gap in the 2.5 km course, which was removed from the analyses. The actual distance covered for each segment was calculated using the elevation difference from the beginning to the end of the segment and the horizontal length of the segment.

Data of speed and time were interpolated by distance for each lap for both Para and AB XC skiers. Further, the average speed and time over the four or six laps were calculated and used in the analyses. In order to compare Para and AB XC skiers, average values of speed and time were calculated for each group of female and male AB XC skiers. Furthermore, the continuous speed and time differences between the male Para and male AB XC skiers and between the female Para and female AB XC skiers were calculated. The proportion of time in the different terrains was calculated for each Para XC skier, the female, and the male AB XC skiers (mean ± standard deviation (SD)).

From the movement data of Para and AB XC skiers, measured by IMUs in the Catapult devices, automatic sub-technique classification was done by employing a K-Nearest Neighbour algorithm while using a 2 s sliding window approach (200 samples) with 95% overlap [15]. The classifier uses the low-pass filtered z-components of the accelerometer and gyroscope data as input, with the z-axis defined in the frontal direction of the participant. The same classifier was used for all skiers. The classifier was validated on AB XC skiers with a per-distance classification accuracy of 96% [6,21,23]. To apply the framework and accompanying algorithms to Para XC skiers with similar accuracy, visual examination of the classification was conducted by comparing the graphical representation of filtered accelerometer and gyroscope signals with examples that typically represent the various sub-techniques. Thereby, around 10% of the cycles from the automatic classification were manually corrected. The sub-techniques were classified as DP, DK, DIA (including both DIA and HRB), and OTHER. OTHER primarily included the tuck position, but also turn techniques and cycles that did not fulfill the above-specified criteria. At higher speeds (i.e., 7 to 10 m·s^−1^), OTHER almost solely contained the tuck position.

### 2.5. Statistical Analysis

In this case-series, descriptive comparison was made for speed, time, and sub-technique distribution between the Para and AB XC skiers, exemplifying the feasibility of the framework based on micro-sensor technology employed in the field for Para XC skiing. Data processing and calculations were done using MATLAB R2018a (version 9.7.0.1190202, MathWorks, Natick, MA) and Microsoft Office Excel 2016 (Microsoft Corporation, Redmond, WA, USA).

## 3. Results

### 3.1. Performance

**Figure 3:**
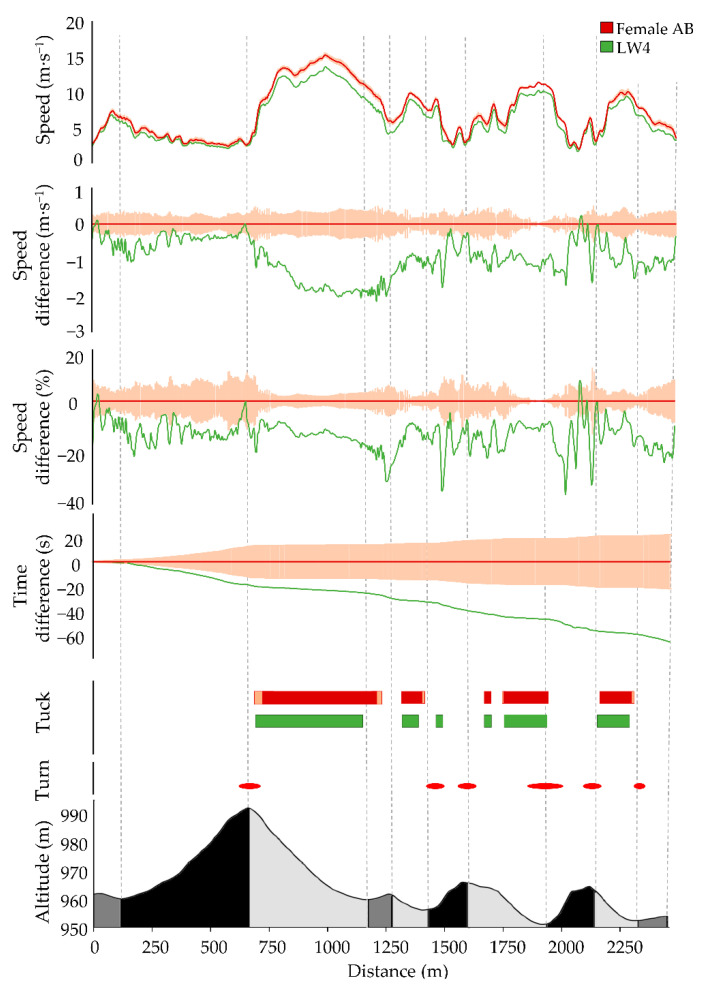
Comparison of average female AB XC skiers (dark red; standard deviation light pink) and female LW4 XC skier (green) for the four laps during the race with respect to average speed, absolute speed difference, relative speed difference, accumulated time difference, and tuck. Course details are visualized in the lower part of the figure; turns (red dashes) and altitude profile of the 2.5 km race course, with uphill (black), flat (dark gray), and downhill (light gray) segments.

The general speed fluctuation patterns of Para and AB XC skiers were similar throughout the race, although Para XC skiers consistently competed at slower speed (Figure 2 and Figure 3, Table 2). Accordingly, the female LW4 skier was 4:26 min slower compared to the female AB XC skiers across the entire race, whereas the male B3a skier was 7:21 min slower and the male B3b skier 12:47 min slower compared to the male AB XC skiers. Compared to male/female AB skiers, male/female Para skiers displayed 19/14% slower average speed with the largest time loss (65 ± 36/35 ± 6 s/lap) found in uphill terrain (Table 2). The relative speed difference between the Para and AB XC skiers was highest in uphill and flat terrain, followed by downhill terrain (Table 2).

### 3.2. Sub-Technique Distribution

**Figure 4:**
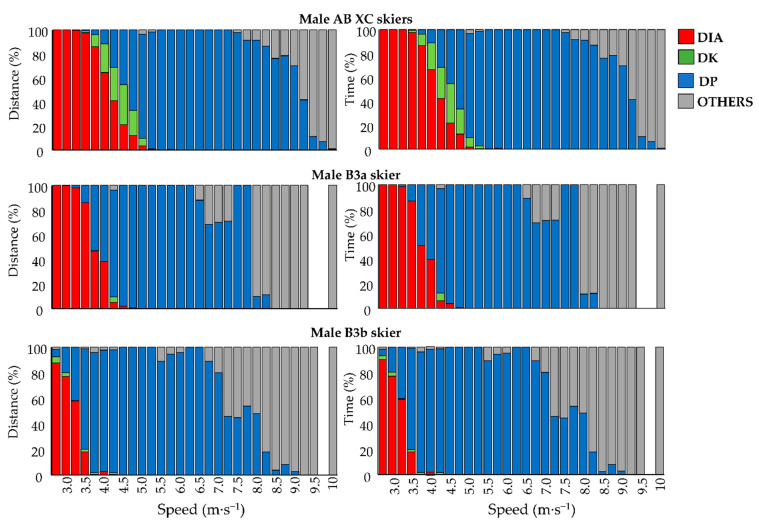
Distribution of sub-techniques over different speed-intervals for male AB, the B3a, and the B3b XC skiers per distance and time. Diagonal stride (DIA; red); Kick double poling (DK; green); Double poling (DP; blue); Tuck position and turn technique (OTHER; gray); White sections illustrate that the skier did not use these speeds.

**Figure 5:**
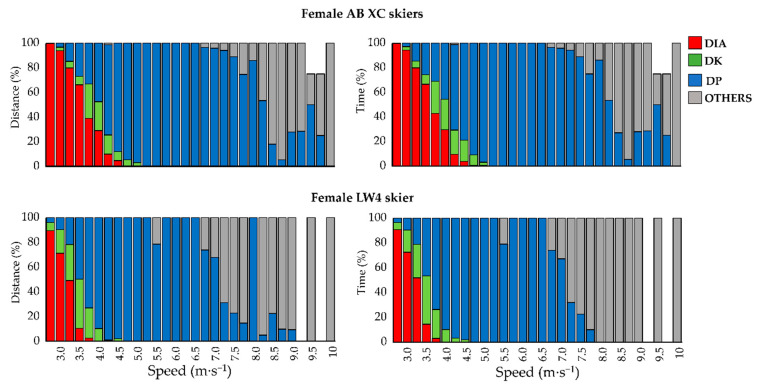
Distribution of sub-techniques over different speed-intervals for female AB and the LW4 XC skiers per distance and time. Diagonal stride (DIA; red); Kick double poling (DK; green); Double poling (DP; blue); Tuck position and turn technique (OTHER; gray). Blank sections for AB XC skiers and the LW4 XC skier illustrate that one of the skiers didn’t used these speeds.

The female Para/AB XC skiers utilized on average DP, DK, DIA, and OTHER, 61/43%, 15/10%, 25/47%, and 0/0% of the distance at lower speed ranges (i.e., 2.75 to 4.75 m·s^−1^), respectively, while the corresponding numbers for male Para/AB XC skiers were 58/18%, 1/13%, 40/69%, and 1/0%. The female Para/AB XC skiers utilized on average DP and OTHER (i.e., tuck position), 26/52% and 74/48% of the distance at higher speed ranges (i.e., 7 to 10 m·s^−1^), respectively, while the corresponding numbers for male Para/AB XC skiers were 29/66% and 71/34% (Figure 4 and Figure 5).

The male B3 and female LW4 XC skiers used the tuck position at similar positions during the race course as the male and female AB XC skiers (Figure 2 and Figure 3) but employed them at a slower speed compared to the AB XC skiers (Figure 4 and Figure 5).

## 4. Discussion

The framework based on micro-sensor technology allowed us to descriptively compare performance and sub-technique selection between standing Para and AB XC skiers in a classical XC skiing race. Using case-series, we revealed that male/female Para skiers displayed 19/14% slower average speed with the largest time loss (65 ± 36/35 ± 6 s per lap) in uphill terrain compared to their AB counterparts. Furthermore, the Para XC skiers utilized a larger proportion of DP than DIA and DK per distance at low speeds (i.e., 2.75 to 4.75 m·s^−1^) and a larger proportion of tuck than DP per distance at high speeds (i.e., 7 to 10 m·s^−1^). Since we are able to distinguish clear differences between Para and AB XC skiers, we propose that the framework is feasible for future use in large-scale investigations of performance at international competitions. Especially, this is the case for more in-depth investigations on the effect of terrain and external conditions on the time factor across Para XC skiing classes.

In line with both the large speed difference and the amount of skiing time spent in uphill terrain (women: LW4: 57% and AB: 58 ± 16%; men: B3: 57 ± 3% and AB: 54 ± 14%), the time loss between the Para and AB XC skiers of the same sex were largest in this terrain. This is in accordance with previous studies in AB XC skiers who spent ~50% of skiing time in uphill terrain, in which the largest performance differences were found [4,19,24,25]. Interestingly, the relative speed difference between Para and AB XC skiers of the same sex in flat terrain was relatively similar to the difference found in uphill terrain. This differs from AB XC skiing, where the relative speed difference is less in flat compared to uphill terrain among different level skiers [19,24,25]. The large relative speed difference in flat terrain may be caused by a reduced balance and motor control of both the female LW4 [26] and the male B3 [26,27] XC skiers due to their impairments. This could have impacted the movement patterns on flat terrain at high speeds.

This is the first study to perform automatic sub-technique classification in standing Para XC skiers during an entire XC skiing race. The comparison to AB XC skiers revealed that, Para XC skiers utilized different proportions of the various sub-techniques at given low and high absolute speeds. This differs from a previous comparison between female and male AB XC skiers during a classical 10 km XC skiing race, that revealed similar proportions of used sub-techniques at same absolute speeds, despite a slower average speed employed by the female AB XC skiers [15]. Regarding the different sub-techniques between Para and AB XC skiers found here, this may be caused by the different (and possibly less technically demanding) coordination stability between arms and legs in DP compared to DIA and DK [28]. Accordingly, the greater use of DP than DIA by the Para XC skiers compared to AB XC skiers at low speeds could be speculated to be caused by the fact that DP is more suitable than DIA and DK for the Para XC skiers in the investigated speeds. In addition, the leg thrust time (i.e., the time during which the ski is in contact with the ground in a leg stride in DIA and DK) in AB XC skiing is suggested to have a speed limit that triggers the transition from DIA to DP [12,29]. For Para XC skiers such limits may be present at a lower speed. Furthermore, the Para XC skiers used a larger proportion of the distance in tuck position than DP at high speeds. Altogether, this indicates that different speed thresholds are present for the choice of classical sub-techniques in Para XC skiers than those suggested by research on AB XC skiers [6,15,17,18]. While this is likely due to disability-related limitations in the Para XC skiers, this still needs further investigation with larger sample sizes during international competitions.

Even though the proposed framework seems feasible for investigating performance also in Para XC skiers, it has some methodological limitations. The framework has only been tested in one race course and under the given external conditions. Since different external conditions (e.g., race courses with and without trees or other obstacles, weather conditions, low- vs. high-speed race courses, etc.) can affect the accuracy of the GNSS receiver, athletes who train and compete in different environments should take this into account and future studies should test the feasibility of the framework under different external conditions. Furthermore, the framework used for automatic sub-technique classification should, in future studies, be adapted for all sitting and standing Para XC skiing categories and validated in larger populations. In line with this, the framework was only applied among standing physically and visually impaired Para XC skiers in the current study, and can, therefore, only be regarded feasible in these athletes.

### Practical Applications

The framework can be used to provide information on where and why Para XC skiers lose or win time compared to their competitors in a race course, as well as the effect of terrain and external conditions on the time factors used in the classification process of Para XC skiers. This could help Para XC skiers to individually define targeted training and competition strategies. In addition, our approach can be used on larger groups of Para XC skiers to provide a more detailed understanding on the influence of sub-technique and terrain on the differences between disabilities, categories, and sexes.

Furthermore, the sub-technique analyses provide information on the specific speeds and terrains where Para XC skiers employ the different sub-techniques, as well as how corresponding temporal patterns within these sub-techniques influence performance. In this study, we found a different distribution of the classical sub-techniques between the standing Para and AB XC skiers both at low and high speeds during an XC skiing race. Together with the large relative speed difference in flat terrain with high skiing speed, the movement pattern of the Para XC skiers seems to be differently exposed to the high speed than AB XC skiers, hence affecting the selected sub-technique. Such information is useful for athletes and coaches when deciding what type of training the different skiers should prioritize (e.g., improvement of technique execution and balance at high skiing speed, or development of aerobic capacity to increase performance in uphill terrain).

## 5. Conclusions

This study evaluated the feasibility of a framework for analyses of performance and sub-technique selection in a heterogenous group of Para XC skiers with different disabilities during a classical XC skiing race. A descriptive comparison of performance and sub-technique selection between Para and AB XC skiers indicated that the largest time loss between the Para and AB XC skiers was found in the uphill terrain. In contrast to the larger speed differences normally found in uphill terrain between performance-levels or sexes within AB skiers, the Para XC skiers displayed a similar relative speed difference compared to AB skiers in flat as in uphill terrain. This may be caused by a reduced balance and motor control of the Para XC skiers due to their impairments and also impact the movement pattern on flat terrain at high speeds. Furthermore, the Para XC skiers more frequently selected DP than DIA and DK at low speeds. Speculatively, DP could be more suitable than DIA and DK due to its lower coordinative demands for Para XC skiers who struggle with stability/coordination. Additionally, the Para XC skiers used a larger proportion of the distance in tuck position than DP at high speeds. Notably, this indicates different speed thresholds of the classical sub-techniques for Para XC skiers compared to AB XC skiers. Altogether, we hypothesize that disability impacts the selection of sub-technique among standing Para XC skiers, which could be examined by using the framework in large-scaled international investigations. Additionally, the framework opens up the possibility to investigate the effect of terrain and external conditions on the time factor across Para XC skiing classes.

## Figures and Tables

**Table 1:** Age, body-mass, and training volume (mean ± SD) of the three Para and seven able-bodied (AB) XC skiers included in the analyses.

Parameter	Paralympic		Able-Bodied	
	Men (*n* = 2)	Woman (*n* = 1)	Men (*n* = 3)	Women (*n* = 4)
Age (years)	24.0 ± 2.8	19.0	25.0 ± 1.5	23.5 ± 1.3
Body-mass (kg)	70.5 ± 1.9	61.0	83.0 ± 2.0	63.5 ± 4.1
Training volume (hours·week^−1^)	13.5 ± 5.0	11.0	16.2 ± 1.0	14.5 ± 1.0

**Table 2:** Proportion of skiing time in different terrain (%), absolute average speed (m·s^−1^), relative speed difference (% of AB XC skiers), and time loss relative to AB XC skiers of same sex per lap (s) for the Para and AB XC skiers.

		LW4	Female AB	B3a	B3b	Male AB
**Proportion** **of time in different terrain (%)**	Uphill	57	58 ± 16	54	59	54 ± 14
	Flat	1.5	14 ± 1	14	14	14 ± 1
	Downhill	28	28 ± 3	31	27	32 ± 3
**Absolute average speed (m·** **s^−1^)**	Overall	6.0 ± 2.3	6.9 ± 2.6	6.7 ±	6.4 ± 2.4	7.9 ± 2.6
	Uphill	3.7 ± 0.5	4.3 ± 0.6	4.6 ± 0.6	3.9 ± 0.5	5.3 ± 0.6
	Flat	5.1 ± 0.7	6.2 ± 1.2	6.0 ± 0.6	5.7 ± 0.8	7.5 ± 1.4
	Downhill	8.3 ± 1.6	9.5 ± 1.9	8.7 ± 1.2	8.9 ± 1.5	10.3 ± 1.9
**Relative speed difference** **(% of AB XC skiers)**	Overall	14 ± 4	100	16 ± 5	20 ± 7	100
	Uphill	14 ± 2	100	14 ± 2	26 ± 3	100
	Flat	16 ± 8	100	19 ± 7	24 ± 4	100
	Downhill	12 ± 1	100	15 ± 4	14 ± 5	100
**Time loss per lap (s)**	Overall	65 ± 10		72 ± 7	126 ± 17	
	Uphill	35 ± 6		13 ± 11	30 ± 24	
	Flat	11 ± 3		4 ± 1	5 ± 1	
	Downhill	19 ± 2		6 ± 4	5 ± 3	
